# MED13L-related disorder characterized by severe motor speech impairment

**DOI:** 10.21203/rs.3.rs-4790993/v1

**Published:** 2024-08-28

**Authors:** Marissa Weyer Mitchel, Stefanie Turner, Lauren K. Walsh, Rebecca I. Torene, Scott M. Myers, Cora M. Taylor

**Affiliations:** Autism & Developmental Medicine Institute Geisinger; Autism & Developmental Medicine Institute Geisinger; Autism & Developmental Medicine Institute Geisinger; Geisinger; Autism & Developmental Medicine Institute Geisinger; Autism & Developmental Medicine Institute Geisinger

**Keywords:** MED13L, Apraxia of speech, Dysarthria, Speech disorders, Motor impairment

## Abstract

**Background:**

*MED13L*-related disorder is associated with intellectual disability, motor delay, and speech deficits. Previous studies have focused on broad clinical descriptions of individuals, but limited information regarding specific speech diagnoses and results of direct testing has been published to date. We conducted deep phenotyping to characterize the speech, language, motor, cognitive, and adaptive phenotypes of individuals with *MED13L*-related disorder.

**Methods:**

In this cross-sectional study, we administered standardized articulation, language, motor, and cognitive testing to 17 children and adolescents (mean age 9y 9m; SD 4y 5m; range 4y 2m to 19y 7m). In-person testing was supplemented with broad developmental, medical, and behavioral information collected virtually from a cohort of 67 individuals.

**Results:**

All individuals who completed in-person articulation testing met diagnostic criteria for speech apraxia, dysarthria, or both. Language impairment was present in all of the in-person cohort and almost all (97%) of the virtual cohort. Those who were able to complete motor testing demonstrated significant deficits in visual motor integration (mean 57.08, SD 9.26). Full scale IQs fell in the borderline to intellectual disability range, consistent with reported cognitive impairment in 97% of the virtual cohort. Notable medical features included hypotonia (83%), vision problems (72%), recurrent otitis media (58%), gastrointestinal problems (57%), and seizures (31%).

**Conclusions:**

*MED13L*-related disorder is characterized by a high rate of motor speech disorders that occur in the context of globally impaired motor, language, and cognitive skills. Children would benefit from intensive, individualized speech therapy and the early adoption of augmentative communication strategies.

## BACKGROUND

The *MED13L* (Mediator Complex Subunit13-like) gene encodes one component of the Mediator transcription co-activator complex, which serves as a bridge between RNA polymerase II and transcription factors (1, 2). It is highly expressed in the fetal and adult brain (particularly the cerebellum), skeletal muscle, and heart (3). Knockdown of *MED13L* deregulates genes within the Wnt signaling pathway, among others, and leads to the impaired development of neural crest cells (1, 2).

Initially, rare heterozygous missense variants in *MED13L* were identified in several patients with cardiac defects (dextra-looped transposition of the great arteries) (3). A later study also identified conotruncal heart defects and intellectual disability in patients with haploinsufficiency of *MED13L* (4). Subsequently, more than 100 patients with disease-causing variants in the *MED13L* gene have been described. Loss of function variants, including whole gene or intragenic deletions, are most common, although there have been recent reports of missense variants associated with higher rates of epilepsy and a more severe phenotype (5, 6). Pathogenic variants almost always occur *de novo*, although there have been three case reports of germline mosaicism (7–9).

Despite the initial reports of heart defects, cardiac anomalies have been reported in a minority of patients with *MED13L* pathogenic variants to date. The most consistently reported features associated with *MED13L*-related disorder are intellectual disability, motor delay, and speech impairment. Additional commonly reported features include facial dysmorphism (macroglossia, macrostomia, depressed nasal root, ear anomalies), hypotonia, and seizure disorder (5, 6, 10). Although severe speech impairment and motor deficits are frequently reported, few specific details regarding the speech and motor phenotype have been provided. The two largest published case series both report severely impaired or absent speech and motor delay in most patients, but do not include results of direct testing or specific speech, language, or motor diagnoses in the majority of individuals (6, 10).

Speech disorders in children can be caused by many factors, but the prevalence and co-occurrence of both speech and motor impairment in *MED13L*-related disorder raises suspicion for possible pediatric motor speech disorders (MSDs), which are rare but intractable speech disorders of childhood (11). The two main pediatric MSDs are childhood apraxia of speech (CAS) and dysarthria. CAS is caused by disrupted motor planning and programming (12), whereas dysarthria arises from deficits in motor execution resulting from impaired muscle strength, tone, or coordination (13). Indeed, there has been one patient who was reported to have a diagnosis of “verbal dyspraxia”(10), an alternative name for CAS, and another case series described one pediatric patient who had been diagnosed with dysarthria (6). However, no detailed phenotypic information or standardized test results were available for these patients beyond general clinical description.

This study examines the speech, language, motor, cognitive, adaptive, and behavioral phenotypes of individuals with *MED13L*-related disorder through detailed articulation, language, psychological, and motor assessments. Deep phenotyping was conducted through in-person testing, supplemented with broad developmental, medical, and behavioral information ascertained from Simons Searchlight (14), a large online cohort of individuals with genetic changes that impact development.

## METHODS

### Patients

#### In-person cohort.

Sixteen individuals were recruited through the 2022 Simons Searchlight Family Meeting, and one additional individual was recruited through an outpatient pediatric neurodevelopmental clinic. Eligibility criteria included age (between 2 and 21 years) and confirmed pathogenic or likely pathogenic (P/LP) *MED13L* variant, interpreted using the American College of Medical Genetics and Genomics/Association for Molecular Pathology (ACMG/AMP) criteria (15). The study was conducted under a minimal risk protocol as defined by the Common Rule, and written, informed consent was obtained for all patients or their legal representatives (45 CFR 46.116).

#### Simons Searchlight cohort.

Sixty-seven individuals with P/LP *MED13L* variants joined the Simons Searchlight study and completed at least some surveys, interviews, and questionnaires. Genetic test reports (exome sequencing or gene panel sequencing) were reviewed for eligibility using ACMG/AMP criteria (15). No individuals with variants of uncertain significance were included. Study data were obtained from a public data clearinghouse, SFARI base (base.sfari.org), with proof of related IRB approval. Thirteen individuals who participated in the in-person protocol also participated in the Simons Searchlight virtual cohort (Table 1).

### In-Person Assessment Protocol

#### Speech.

Fourteen individuals produced enough verbal speech to complete the speech assessment, which was administered by a certified speech-language pathologist with expertise in pediatric MSDs. The Goldman-Fristoe Test of Articulation- Third Edition (GFTA-3) (16) was administered to all 14 individuals and completed in its entirety by 12 children. The Dynamic Evaluation of Motor Speech Skill (DEMSS) (17) was administered to a subset of four children to gather additional information regarding core features of CAS, including inconsistency, disrupted prosody, and vowel errors. A naturalistic speech sample was transcribed for all 14 individuals and analyzed for features of CAS and dysarthria. The Profile of Childhood Apraxia of speech and Dysarthria (ProCAD) (18) was utilized to aid with differential diagnosis of individuals’ speech disorders.

#### Language.

Two standardized language tests, the Peabody Picture Vocabulary Test, Fifth Edition (PPVT-5) (19) and Expressive Vocabulary Test, Third Edition (EVT-3) (20), were administered by a certified speech-language pathologist to evaluate receptive and expressive language skills, respectively.

#### Cognition.

A pediatric psychologist or trained research assistant administered one of three cognitive tests to all individuals, depending on age and ability level: the Differential Abilities Scale, Second Edition (DAS-2); Kaufman Brief Intelligence Test, Second Edition (KBIT-2) (22); or the Mullen Scales of Early Learning (MSEL) (23).

#### Visual Motor.

The Beery-Buktenica Developmental Test of Visual Motor Integration, Sixth Edition (Beery VMI) (24) was administered by a pediatric psychologist or trained research assistant to all individuals and was completed in its entirety by 12 children.

### Virtual Assessment Protocol

#### Medical and Developmental.

Simons Searchlight genetic counselors administered standardized medical and developmental histories by telephone interview with primary caregivers of individuals with *MED13L*-related disorder. A standardized seizure history survey was obtained by online questionnaire. One individual was recruited from a neurodevelopmental clinic and medical history data was ascertained through review of electronic health record (EHR) data.

#### Adaptive.

The Vineland Adaptive Behavior Scales, Third Edition (VABS) (25) was completed by online questionnaire by primary caregivers.

#### Behavior.

The Child Behavior Checklist (CBCL) (26, 27) was completed by online questionnaire by primary caregivers. Two forms of the CBCL were administered based on the age of the child (ages 1.5–5 and ages 6–18).

## RESULTS

### In-person cohort

**Figure 1:**
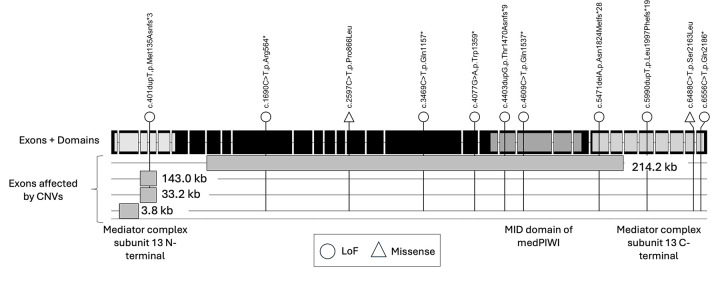
*MED13L* (NM_015335.5) exons, functional domains, and pathogenic variants of in-person cohort were plotted using the trackViewer library.(30) Note that intragenic deletions are shown relative to exons although they also span introns, thus their sizes are not to scale in this figure.

Seventeen individuals (10 female, 7 male) from 15 families participated in the in-person assessment, including two sets of siblings. The mean age was 9 years, 9 months (range 4y 2m to 19y 7m; SD 4y 5m). Sixteen of 17 variants were *de novo*, with inferred germline mosaicism for the two families with affected siblings (Table 1). The single case of an inherited variant was from a father with somatic mosaicism (pathogenic variant identified in 7% of reads). There were 12 sequence variants (10 nonsense/frameshift, 2 missense) and 5 intragenic deletions (Fig. 1).

#### Speech.

Three children did not produce enough verbal speech to complete articulation testing. The remaining 14 who participated in articulation testing met diagnostic criteria for at least one MSD: 7 with CAS only, 6 with CAS + dysarthria, and 1 with dysarthria only (Table 2). On average, individuals exhibited 10 characteristic features of CAS or dysarthria recorded by the ProCAD (range 5–15; SD 2.88) (Table S1, Supplementary Material). Five participants also exhibited a comorbid phonological disorder, which is a non-motor cognitive-linguistic disorder impacting speech production at the phonemic level. Twelve participants completed the GFTA-3, and all individuals’ raw scores corresponded to the lowest achievable standard score (40). Because of this floor effect, growth scale values rather than standard scores are reported in Table 2. Four participants also completed the DEMSS, in which lower scores are associated with higher likelihood of a CAS diagnosis. Participants’ raw scores on this measure ranged from 353 (evidence for at least mild CAS) to 114 (significant evidence for CAS). Seven children used some form of augmentative and alternative communication (AAC), including speech-generating devices (6 children) and sign language (1 child). An additional child had attempted to use AAC but had not been successful. While non-speech oral-motor skills were not measured comprehensively, families reported a high rate of dysfunction in this area, including difficulty feeding in infancy (6 children), drooling (4 children), and open mouth posture/tongue thrust (3 children).

#### Language.

Fourteen participants completed the PPVT-5, and 13 participants completed the EVT-3. All participants scored below the average range on both receptive and expressive vocabulary tests (Table 3). The average standard score on the PPVT-5 was 54.86 (range 40–79; SD 12.04) and the average standard score on the EVT-3 was 60.00 (range 40–80; SD 11.92). Most participants did not exhibit a significant difference between their scores on the PPVT-5 and EVT-3. Out of 17 individuals who participated in at least some aspects of the in-person assessment, 3 children were minimally verbal, 12 children exhibited single-word or phrase-level speech, and 2 children were conversational.

#### Cognition.

Fifteen individuals completed a cognitive assessment. Three individuals completed the MSEL, six individuals completed the DAS-2, and six individuals completed the KBIT-2. Overall, all participants had measured full scale IQs within the range of borderline to intellectual disability (Table 3). The average full scale IQ score was 46.20 (SD 20.20). In cases where both verbal and nonverbal IQ scores were calculable, nonverbal scores (mean 63.00, SD 18.96) were higher than verbal scores (mean 51.18, SD 14.37).

#### Visual Motor.

Twelve individuals with *MED13L* completed the Beery VMI. Three additional individuals attempted but were unable to complete any items on the VMI (would not grasp a pencil or make marks on paper). Of those who completed the Beery VMI, three scored at the floor of the assessment. Overall, all individuals demonstrated significant deficits in visual motor integration (average 57.08, standard deviation 9.26) (Table 3).

### Virtual Cohort

#### Medical and Developmental.

Caregivers of 59 individuals with P/LP pathogenic variants from Simons Searchlight completed the medical history interview, and one additional individual’s medical information was ascertained through EHR review (N = 60; Table S2 in Supplementary Material). Most individuals (58, 97%) were reported to have at least one neurological feature other than seizures, with low muscle tone being the most common (50, 83%). Additional neurological features included clumsiness/coordination disorder (13, 22%), high muscle tone (8, 13%), movement disorder (6, 10%), large head size (6, 10%), small head size (3, 5%), and cerebral palsy (2, 3%). Gastrointestinal problems, including constipation, gastroesophageal reflux, and diarrhea, occurred in over half of patients (34, 57%). Other prevalent medical features included vision problems (43, 72%) and recurrent otitis media (35, 58%). Cardiac anomalies (including patent ductus arteriosis, tetralogy of Fallot, and bicuspid aortic valve, among others) varied widely and occurred in a minority of individuals (11, 18%).

**Figure 2:**
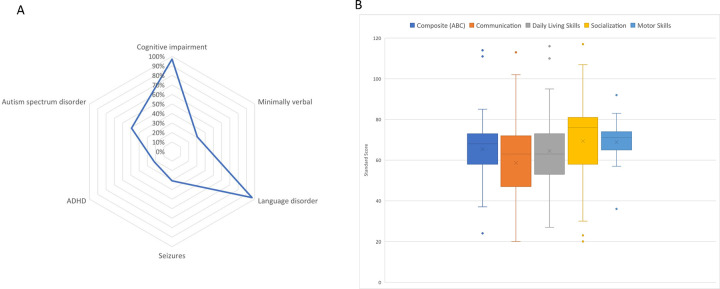
Neurodevelopmental diagnoses and adaptive skills of virtual cohort. A. Radar plot displaying the most common neurodevelopmental diagnoses reported by caregivers of children with *MED13L* pathogenic variants (N = 67). Developmental histories obtained by standardized telephone interview with trained genetic counselors through Simons Searchlight. B. Box and whiskers plot displaying the average standard scores of the Vineland Adaptive Behavior Scale for the four main domains (communication, daily living skills, socialization, and motor skills) and the composite score (N = 47). Mean (X), median, interquartile range, and outlier points are depicted.

Sixty-five individuals’ caregivers completed a separate seizure questionnaire, and 67 completed a developmental history interview (Fig. 2A). Seizures were reported in a sizable minority of patients (20/65, 31%), seven of whom required emergency intervention. All but two individuals (97%) with P/LP *MED13L* variants were reported to have some degree of cognitive impairment, including intellectual disability, developmental delay, and/or learning disability. A similar percentage of individuals also reported a language disorder diagnosis (63/65, 97%), with about a third of children older than 48 months described as minimally verbal (15/49, 31%). Other neurodevelopmental disorders were reported in fewer than half of patients, including autism spectrum disorder (28/57, 49%) and attention deficit/hyperactivity disorder (13/60, 22%).

#### Adaptive.

Caregivers completed the VABS-3 on 47 individuals with pathogenic mutations in *MED13L*. Adaptive scores demonstrate delays across all areas measured and no areas of relative adaptive strengths or weaknesses are present (Fig. 2B; Table S3 in Supplementary Material).

#### Behavior.

Parent report of behavioral symptoms in the 1.5–5-year age range using the CBCL was completed for 31 individuals (Table S4 in Supplementary Material). Results show no mean scores within the clinically elevated range (> 98th percentile) of any scale or subscale. Mean subclinical elevations (95th −98th percentile) were identified in the domains of withdrawal and attention problems. Parent report of behavioral symptoms in the 6–18 year age range using the CBCL was completed on 25 individuals and no mean scores within the clinically elevated range are present (Table S5 in Supplementary Material). Mean subclinical elevations were identified in the domains of social problems, thought problems, and attention problems.

## DISCUSSION

In this study, we completed deep phenotyping of 17 children and adolescents with *MED13L*-related disorder and analyzed medical, developmental, and behavioral data of a larger group of up to 67 individuals. These findings support previous case reports of significant cognitive and adaptive delays, but with a larger cohort. Our study is the first to describe detailed speech and language features in *MED13L*-related disorder. Strikingly, all individuals who completed in-person phenotyping met criteria for a diagnosis of MSD (CAS, dysarthria, or both). Additionally, all individuals who participated in in-person testing exhibited severe receptive and expressive language impairment with commensurate skills in comprehension and production. Almost all individuals (97%) from the full cohort of 67 reported a diagnosis of language disorder, with about a third of children described as minimally verbal or nonverbal beyond the age of 4 years. Together, these results suggest that *MED13L*-related disorder is characterized by pervasive MSD and severe language impairment.

Broad motor developmental delay is another key feature of *MED13L*-related disorder. Direct testing revealed particularly severe fine motor and visual motor difficulties in all individuals assessed, with a subset of children unable to participate in direct testing due to their level of impairment. Results of in-person testing are in line with caregiver report of the larger cohort, describing broad gross and fine motor deficits in most individuals. Interestingly, caregivers reported high rates of muscle tone abnormalities, coordination difficulties, and movement disorder diagnoses, with two individuals reporting a diagnosis of cerebral palsy. Such findings suggest that MSDs in this group of children are not isolated diagnoses; rather, they occur in the context of globally impaired motor planning and execution.

Other notable findings were the absence of clinically significant internalizing or externalizing behavior problems and relatively low rates of comorbid neuropsychiatric disorders, such as attention deficit/hyperactivity disorder. This is surprising given previous research demonstrating higher rates of behavioral and psychiatric disorders among individuals with intellectual disability compared to the general population (28). While several subscales of the CBCL did indicate some increased behavioral concerns, there were no identifying behavioral phenotypes. Another unexpected observation was the identification of seven individuals from four unrelated families with P/LP *MED13L* variants due to presumed germline mosaicism. At least three unrelated individuals with pathogenic *MED13L* variants because of parental germline mosaicism have been published to date, (7–9), bringing the total number to seven families in the literature with presumed or confirmed mosaicism. This is higher than the traditionally reported 1–2% recurrence risk for parents after identification of a *de novo* variant in their child, but it may be in line with more recent studies suggesting that individualized recurrence risk may be higher for some families (29). Given the seemingly high prevalence of parental mosaicism in *MED13L*-related disorder, additional research is warranted to systematically assess the prevalence and recurrence risks associated with this condition.

A limitation of this study is that all participants were ascertained through clinical genetic testing and may not represent the full range of the phenotypic spectrum of *MED13L*-related disorder. It is possible that more mildly affected individuals with *MED13L* variants may not have routine access to genetic testing and therefore would not be included in our study. Although beyond the scope of this study, future population studies examining the true prevalence and phenotypic spectrum of *MED13L* variants would be valuable. Another limitation is that standardized scores for participants who completed in-person testing could be calculated only for the subset of individuals who were able to complete testing. Because individuals with the most severe speech and motor disorders were often unable to participate fully, the average standard scores reported here are likely skewed and may not accurately reflect the lower range of ability in *MED13L*-related disorder.

The high rate of severe MSD, including a sizable minority of children who are minimally verbal beyond age four, has clear implications for treatment planning with this population. Children with known P/LP variants in *MED13L* should be provided access to AAC strategies, including speech-generating devices, from a young age. In our study, even children and adolescents who were verbal benefitted from speech-generating devices due to poor speech intelligibility resulting from their MSD. Further, there should be a low threshold for suspicion of MSD in children with *MED13L*-related disorder, and evidence-based motor learning techniques should be incorporated into speech therapy. Comprehensive evaluation of fine and gross motor skills is also recommended so that appropriate occupational and physical therapies can be provided.

These results suggest that *MED13L*-related disorder is characterized by intellectual disability, global motor impairment, and a severe, superimposed MSD phenotype. Other behavioral and neuropsychiatric diagnoses are relatively uncommon in this population. Despite early reports of cardiac defects associated with *MED13L* variants, congenital heart anomalies occurred infrequently in this cohort. Future research with a larger population is needed to investigate the relationship between genotype and speech/motor phenotype. Additional, comprehensive gross and fine motor testing would also be helpful to delineate granular aspects of motor planning, programming, and execution among individuals with *MED13L*-related disorder.

## CONCLUSIONS

*MED13L*-related disorder is characterized by a high rate of motor speech disorders, including apraxia and dysarthria, that occur in the context of globally impaired motor, language, and cognitive skills. These results suggest that children with known P/LP variants in *MED13L* would benefit from intensive, individualized speech therapy and the early adoption of augmentative communication strategies.

## Figures and Tables

**Table 1:** MED13L variants of the in-person cohort

ID	Variant (NM_015335.4)	Type	Inheritance	Classification
F1S1^a^	c.5990dup,p.Leu1997Phefs*19	LoF	De novo	Pathogenic
F2S1	c.401dup,p.Met135Asnfs*3	LoF	De novo	Pathogenic
F3S1^a^	c.5471del,p.Asn1824Metfs*28	LoF	Inherited - Paternal (somatic mosaic; 7% of reads)	Pathogenic
F4S1^a^	c.2597C > T,p.Pro866Leu	Missense	De novo	Pathogenic
F5S1^a^	arr[GRCh37] 12q24.21(116519929–116553225)x1	Deletion	Unknown	Pathogenic
F6S1^a^	c.6556C > T,p.Gln2186*	LoF	De novo	Likely Pathogenic
F7S1^a^	c.3469C > T,p.Gln1157*	LoF	De novo	Pathogenic
F8S1^a^	arr[GRCh37] 12q24.21(116,673,140 – 116,676,995)x1	Deletion	De novo (germline mosaicism inferred)	Pathogenic
F8S2^a^	arr[GRCh37] 12q24.21(116,673,140 – 116,676,995)x1	Deletion	De novo (germline mosaicism inferred)	Pathogenic
F9S1	C.4077G > A,p.Trp1359*	LoF	De novo	Pathogenic
F10S1	arr[GRCh37] 12q24.21(116476163_116619234)x1	Deletion	De novo	Pathogenic
F11S1^a^	arr[hg18] 12q24.21(114,895,862 – 115,110,038)x1	Deletion	De novo	Pathogenic
F12S1^a^	c.4609C > T,p.Gln1537*	LoF	De novo	Pathogenic
F13S1^a^	c.4403dup,p.Thr1470Asnfs*9	LoF	De novo	Pathogenic
F14S1^a^	c.1690C > T,p.Arg564*	LoF	De novo (germline mosaicism inferred)	Pathogenic
F14S2^a^	c.1690C > T,p.Arg564*	LoF	De novo (germline mosaicism inferred)	Pathogenic
F15S1	c.6488C > T,p.Ser2163Leu	Missense	De novo	Likely Pathogenic

a Individuals also completed some aspects of Simons Searchlight virtual assessment protocol.

Abbreviations: LoF = loss of function

**Table 2:** Speech and oral-motor phenotype

ID	CAS	Dysarthria	Phonological disorder	GFTA-3 growth scale value	DEMSS raw score	Oral-motor dysfunction	AAC
F1S1	+	-	+	452	NA	-	-
F2S1	+	-	-	NA	114	-	Uses SGD
F3S1	+	+	-	460	NA	Drooling	-
F4S1	NA	NA	NA	NA	NA	Drooling; open mouth posture; difficulty feeding in infancy	Uses SGD
F5S1	+	+	-	507	NA	-	-
F6S1	+	-	+	472	NA	Oral hypotonia; open-mouth posture	
F7S1	NA	NA	NA	441	NA	-	Attempted but not successful
F8S1	+	-	-	465	NA	-	Uses SGD
F8S2	NA	NA	NA	515	356	Difficulty feeding in infancy	Uses SGD
F9S1	-	+	+	497	NA	-	Uses sign language
F10S1	+	-	-	515	353	Difficulty feeding in infancy	-
F11S1	+	+	-	491	NA	-	-
F12S1	+	+	-	481	358	Drooling; tongue thrust; difficulty feeding in infancy	
F13S1	+	+	-	441	NA	Difficulty feeding in infancy	-
F14S1	+	-	+	452	NA	Drooling	Uses SGD
F14S2	+	-	+	NA	114	-	-
F15S1	+	+	-	460	NA	Difficulty feeding in infancy, history of dysphagia	Uses SGD

Abbreviations: CAS = childhood apraxia of speech; GFTA-3 = Goldman Fristoe Test of Articulation, 5th Edition; DEMSS = Dynamic Evaluation of Motor Speech Skills; AAC = augmentative and alternative communication; SGD = speech-generating device

**Table 3:** Cognitive, language, visual-motor testing standard scores

ID	Age in months	FSIQ	Verbal IQ	Nonverbal IQ	PPVT-5	EVT-3	Beery VMI
F1S1	97	U	U	U	62	58	U
F2S1	73	64	43	85	56	50	65
F3S1	58	67	57	77	62	65	56
F4S1	67	49	20	20	50	U	U
F5S1	213	40	42	44	40	50	45
F6S1	94	48	46	69	40	67	58
F7S1	88	10^a^	U	U	U	U	U
F8S1	134	20^a^	U	U	40	40	U
F8S2	154	8^a^	U	U	51	67	45
F9S1	116	35^a^	U	U	U	U	56
F10S1	50	54	45	66	U	U	72
F11S1	155	54	63	56	59	67	57
F12S1	67	M	M	M	79	80	M
F13S1	160	68	65	79	58	69	63
F14S1	138	52	60	55	62	60	70
F14S2	99	72	72	80	69	67	53
F15S1	235	52	50	62	40	40	45
**Mean**	117.53	46.20	51.18	63.00	54.86	60.00	57.08
**SD**	53.34	20.20	14.37	18.86	12.04	11.92	9.27

a IQ extrapolated from age equivalents on developmental assessment subtests. Abbreviations: FSIQ = full scale IQ; PPVT-5 = Peabody Picture Vocabulary Test, 5th Edition; EVT-3 = Expressive Vocabulary Test, 3rd Edition; Beery VMI = Beery-Buktenica Test of Visual-Motor Integration; U = patient unable to complete; M = missing data/test not administered.

**Additional Files**

File Name: Additional File 1.xlsx

Description of Data

## Data Availability

Deidentified participant data from Simons Searchlight used in this study can be obtained by request (http://base.sfari.org). Reuse of data is permitted on submission of a data request. Additional data that support the findings of this study are available from the corresponding author upon reasonable request.

## Abbreviations

MSD: Motor speech disorder
CAS: Childhood apraxia of speech
P/LP: Pathogenic/Likely pathogenic
ACMG: American College of Medical Genetics and Genomics
AMP: Association for Molecular Pathology
GFTA-3: Goldman Fristoe Test of Articulation, Third Edition
DEMSS: Dynamic Evaluation of Motor Speech Skill
ProCAD: Profile of Childhood Apraxia of Speech and Dysarthria
PPVT-5: Peabody Picture Vocabulary Test, Fifth Edition
EVT-3: Expressive Vocabulary Test, Third Edition
DAS-2: Differential Abilities Scale, Second Edition
KBIT-2: Kaufman Brief Intelligence Test, Second Edition
MSEL: Mullen Scales of Early Learning
Beery: VMI Beery-Buktenica Developmental Test of Visual Motor Integration
HER: Electronic health record
VABS: Vineland Adaptive Behavior Scale
CBCL: Child Behavior Checklist
AAC: Augmentative and alternative communication
